# AMP-Activated Protein Kinase-Regulated Activation of the PGC-1α Promoter in Skeletal Muscle Cells

**DOI:** 10.1371/journal.pone.0003614

**Published:** 2008-10-31

**Authors:** Isabella Irrcher, Vladimir Ljubicic, Angie F. Kirwan, David A. Hood

**Affiliations:** 1 School of Kinesiology and Health Science, York University, Toronto, Ontario, Canada; 2 Muscle Health Research Centre, York University, Toronto, Ontario, Canada; 3 Department of Biology, York University, Toronto, Ontario, Canada; Universidad Europea de Madrid, Spain

## Abstract

The mechanisms by which PGC-1α gene expression is controlled in skeletal muscle remains largely undefined. Thus, we sought to investigate the transcriptional regulation of PGC-1α using AICAR, an activator of AMPK, that is known to increase PGC-1α expression. A 2.2 kb fragment of the human PGC-1α promoter was cloned and sequence analysis revealed that this TATA-less sequence houses putative consensus sites including a GC-box, a CRE, several IRSs, a SRE, binding sites for GATA, MEF2, p 53, NF-κB, and EBox binding proteins. AMPK activation for 24 hours increased PGC-1α promoter activity with concomitant increases in mRNA expression. The effect of AICAR on transcriptional activation was mediated by an overlapping GATA/EBox binding site at −495 within the PGC-1α promoter based on gel shift analyses that revealed increases in GATA/EBox DNA binding. Mutation of the EBox within the GATA/EBox binding site in the promoter reduced basal promoter activity and completely abolished the AICAR effect. Supershift analyses identified USF-1 as a DNA binding transcription factor potentially involved in regulating PGC-1α promoter activity, which was confirmed *in vivo* by ChIP. Overexpression of either GATA-4 or USF-1 alone increased the p851 PGC-1α promoter activity by 1.7- and 2.0-fold respectively, while co-expression of GATA-4 and USF-1 led to an additive increase in PGC-1α promoter activity. The USF-1-mediated increase in PGC-1α promoter activation led to similar increases at the mRNA level. Our data identify a novel AMPK-mediated regulatory pathway that regulates PGC-1α gene expression. This could represent a potential therapeutic target to control PGC-1α expression in skeletal muscle.

## Introduction

Skeletal muscle exhibits remarkable plasticity in response changing energy demands. For example, repeated bouts of exercise in the form of endurance exercise training of an appropriate time, duration and intensity can induce mitochondrial phenotype and content changes within muscle cells, a process termed mitochondrial biogenesis. This adaptation is associated with numerous clinical and health related benefits including improvements in oxidative capacity [1], exercise tolerance [2], the alleviation of symptoms associated with physical inactivity-related diseases such as insulin resistance [3], as well as the possible attenuation of the decline in oxidative capacity associated with aging [4]. Mitochondrial biogenesis is controlled via the actions of numerous transcription factors and transcriptional co-activators. This serves to coordinate the nuclear and mitochondrial genomes, and ultimately plays an important role in regulating the stoichiometric production and assembly of the proteins involved in organelle synthesis [5].

Recently, the transcriptional co-activator PPARγ-coactivator-1 protein α (PGC-1α) has been proposed to play a central role in regulating mitochondrial content within cells [6], [7]. PGC-1α is induced by mitochondrial biogenesis-inducing stimuli such as thyroid hormone treatment, as well as contractile activity *in vivo* and *in vitro* in skeletal muscle [8], [9], [10]. Moreover, low levels of PGC-1α expression in muscle have been associated with defects in energy metabolism, in addition to reduced mitochondrial content and function [11], [12]. The importance of PGC-1α in regulating mitochondrial content and function suggests that further investigation into the regulation of PGC-1α gene expression is warranted particularly under conditions in which mitochondrial biogenesis is induced. In recent years, several signaling kinases have been implicated in mediating the transcriptional activation of the PGC-1α promoter activity and mRNA expression in response to various stimuli [13]–[17] suggesting that PGC-1α gene expression is controlled, in part, at a transcriptional level. The signaling events associated with the induction of mitochondrial biogenesis and increases in PGC-1α gene expression within skeletal muscle remain largely undefined.

In skeletal muscle, numerous signaling kinases involved in initiating mitochondrial biogenesis have been described including the activation of AMP-kinase (AMPK). A decrease in the ratio of ATP/AMP within muscle cells activates AMPK [18], [19]. Pharmacological activation of AMPK using 5-aminoimidazole-4-carboxamide-1-β-D-ribofuranoside (AICAR) stimulates mitochondrial biogenesis, and this is likely to occur through the induction of PGC-1α [9], [18]. AMPK is also activated by exercise in rodents [20], humans [21], [22] and following electrical stimulation of skeletal muscle [9], [23], stimuli which are known to induce mitochondrial biogenesis. Since AMPK is likely a key signaling molecule in the pathway leading to mitochondrial biogenesis in skeletal muscle, we sought to investigate the potential role of AMPK in regulating PGC-1α expression via transcriptional activation of its promoter. Here we report the characterization of the human PGC-1α promoter in skeletal muscle cells, and examine its regulation following activation of AMPK via AICAR. Furthermore, we identify potential AMPK transcription factor targets that mediate increases in PGC-1α transcription in muscle.

## Results

### Characterization of the proximal 2 kb human PGC-1α promoter

**Figure 1:**
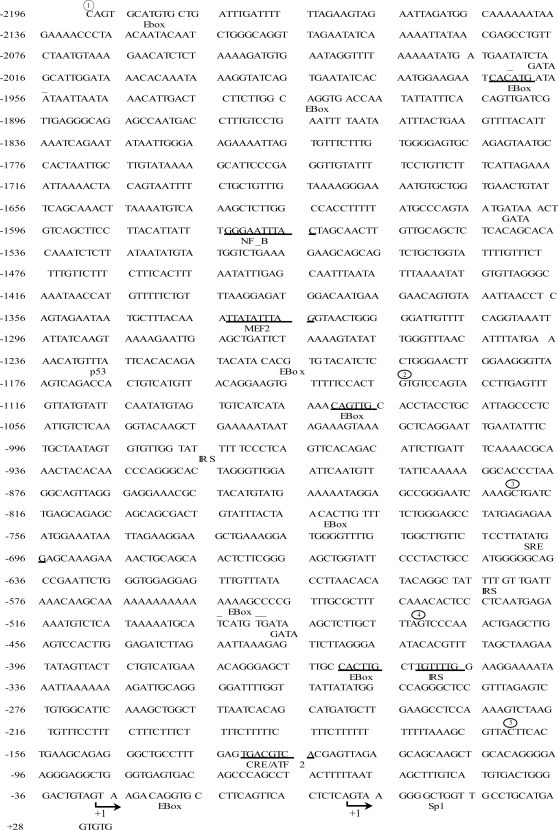
The human PGC-1α promoter. The nucleotide sequence +28 to −2190 corresponding to the proximal 2-kb hPGC-1α promoter is shown. The arrows indicate the transcription start sites, which have been previously described [14]. Putative binding sites for transcription factors are either *underlined* or *overlined*. Also included are binding sites for transcription factors that have previously been characterized [12;13;19]. Numbers enclosed in circles represent the 5′- deletions of the PGC-1α promoter reporter constructs 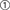 p 2215, 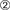 p 1164, 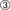 p 851, 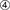 p501, 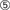 p 191 as shown in Fig. 2.

The mechanism(s) regulating PGC-1α transcription were first investigated by cloning the proximal 2 kb sequence of the human PGC-1α promoter. This sequence contains +28 to −2190 nucleotides relative to the first transcriptional start site (GenBank Accession No. BD103728; [24]. Inspection of this sequence for the presence of consensus transcription factor binding sites was performed by high stringency searches using PATCH (Pattern search for transcription factor binding sites) and TRANSFAC 6.0. The odds of identifying false positives were minimized by excluding non-canonical sequences, or sequences that contained nucleotide mismatches. The putative DNA binding sites that were found within the hPGC-1α promoter are identified in Fig. 1. Although the promoter does not appear to contain a TATA box, our search identified a putative GC Box within the first intron that has been shown to bind Sp1 [25]. This finding is also in line with those of Esterbauer *et al* [24]. In addition to the putative Sp1 site, there is one consensus cAMP Response Element (CRE) at position −133, three insulin response sequences (IRS) at −354, −589, and −979, and one Muscle Enhancer Factor-2 (MEF2) site at −1335. These sequences have recently been shown to bind the cAMP Response Element Binding protein (CREB) and a CREB-related family member, Activating Transcription Factor 2 (ATF2), the forkhead transcription factor (FKHR) and MEF2, respectively [15], [16], [26]. Our search also identified three GATA sequences at −493, −1605, and −1961, as well as a consensus sequence representing the serum response element (SRE) at −705 upstream from the transcriptional start site. Putative binding sites for p53 at −1237 and NF-kB at −1575, as well as multiple EBoxes at −23, −362, −495, −785, −1083, −1190, −1927, −1995 and −2185 were also found within the PGC-1α promoter.

### Effect of AICAR on AMPK activation and PGC-1α transcription

**Figure 2:**
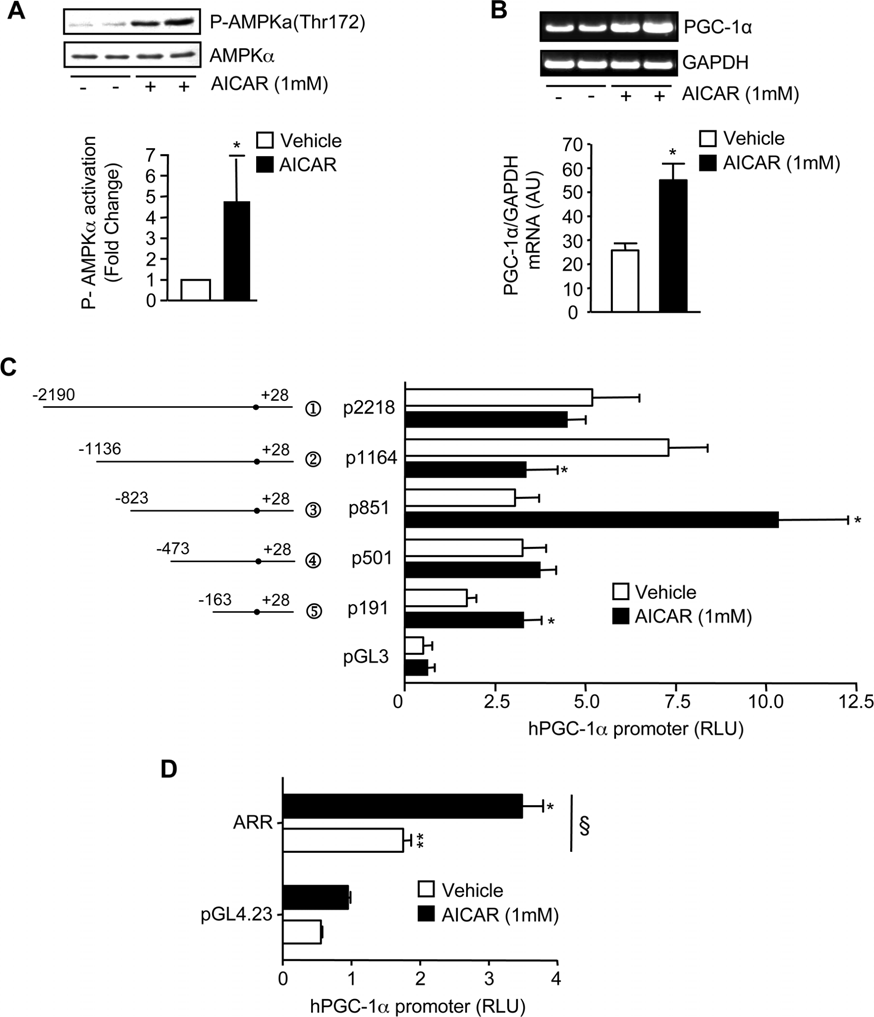
AMPK activation induces PGC-1α mRNA expression and transcriptionally activates the PGC-1α promoter. C_2_C_12_ cells were treated with either AICAR (1 mM) or Vehicle for 24 hrs. A. Representative Western Blot probed with a Phospho-AMPKα (Thr172), stripped and then re-probed with total AMPKα for loading control (upper panel). Summary of repeated experiments of the effect of AICAR on AMPK activation is shown (lower panel; n = 4). B. upper panel, EtBr-stained DNA gel of PGC-1α amplified by PCR from vehicle- and AICAR-treated cells. GAPDH was also amplified by PCR and used to verify equal loading. Lower panel: A summary of repeated experiments of the effects of AICAR on PGC-1α mRNA expression (n = 3). C. AICAR-induced transcriptional regulation of the PGC-1α promoter. Relative luciferase activity of the PGC-1α promoter constructs in vehicle- or AICAR-treated cells is shown (n = 4–6). D. The AICAR-responsive region (ARR) from −473 to −821 was cloned into the pGL4.23 minimal promoter vector and AICAR-induced transcriptional regulation of this region was assessed (n = 3). For all data, values are means±S.E.M, *, p<0.05 versus Vehicle-treated control; §, p<005 versus pGL4.23.

AMPK activation via AICAR treatment has previously been shown to upregulate PGC-1α mRNA and protein expression in skeletal muscle [9], [27]–[29]. Thus, we used AICAR to investigate the AMPK-mediated transcriptional control of PGC-1α in C_2_C_12_ cells. Following 24 hours of treatment with AICAR, AMPK activation via phosphorylation on Thr172 was increased by 4.5-fold (p<0.05; Fig. 2A). This occurred concomitantly with a 2.2- fold increase (p<0.05) in PGC-1α mRNA expression (Fig. 2B). Next, a series of 5′-deletions of the 2 kb PGC-1α promoter were generated and transfected into cells to determine whether this was related to increased transcriptional activity. The responsiveness of the promoter-luciferase reporter constructs to AMPK activation was assessed after 24 hours of treatment with 1 mM AICAR. A two-way ANOVA was performed and a significant main effect of promoter length was found. Post-hoc tests revealed significant 3.5- and 1.8-fold increases (p<0.05) in the p 851 and p191 promoters respectively, as well as a 2.2-fold decrease (p<0.05) in the p1164 promoter (Fig. 2C). To evaluate whether the −823 to −473 AICAR-responsive region (ARR) is sufficient to activate a minimal promoter, a construct containing this region was cloned upstream of a minimal promoter in the pGL4.23 reporter vector. Two-way ANOVA revealed a significant interaction (p<0.05) between the ARR and the effect of AICAR (Fig. 2D). Post-hoc tests indicated a significant 3.1- fold increase (p<0.05) in basal promoter activity. This activity of the AICAR-responsive region was further increased 2-fold (p<0.05) in the presence of AICAR.

### Effect of AMPK activation on DNA binding and protein expression

**Figure 3:**
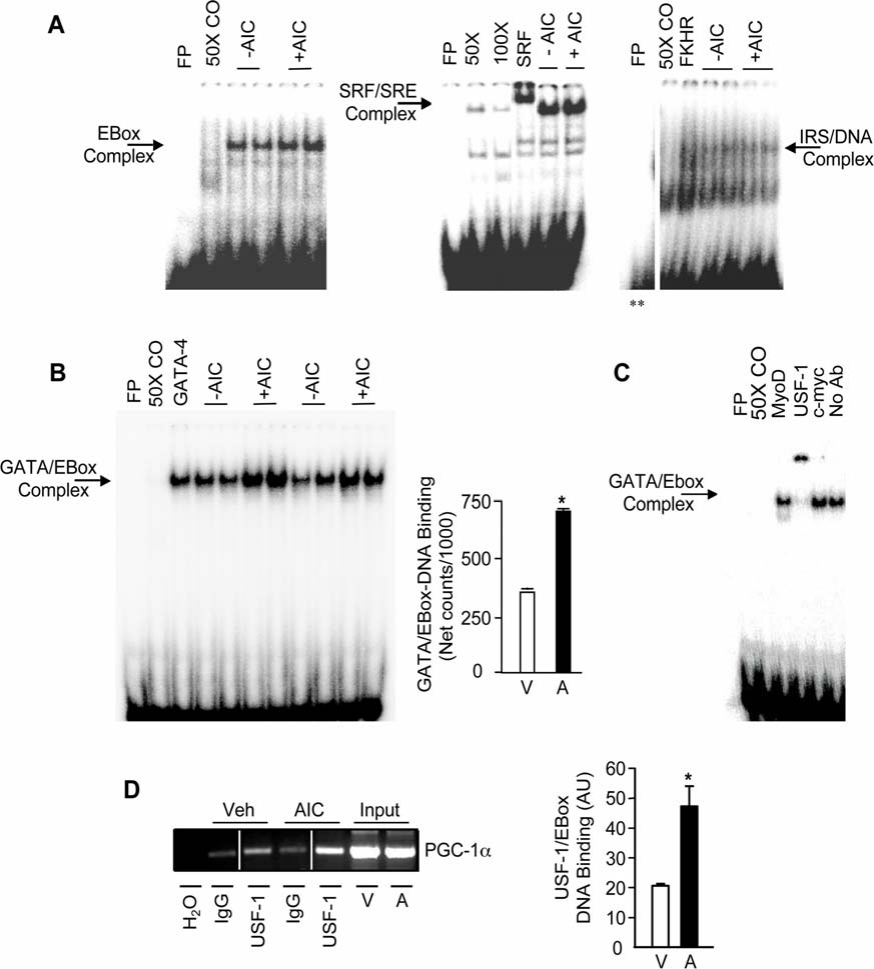
AMPK activation increases GATA/EBox DNA binding. A. Representative EMSAs of nuclear extracts from Vehicle− (−AIC) and AICAR− (+AIC) treated cells that were incubated with radiolabeled oligonucleotides corresponding to the EBox, SRE and IRS sequences found within the AICAR-responsive region of the PGC-1α promoter (as shown in Fig. 1). B. A representative EMSA (*Left panel)* and a summary of multiple experiments (*Right panel)* showing the effect of AICAR (A) on GATA/EBox-DNA binding. Values are represented as means±S.E.M (n = 8) relative to vehicle-treated (V) cells. C. Representative EMSAs of nuclear extracts that were incubated with radiolabeled oligonucleotides corresponding to GATA/EBox wt. Vehicle-treated cells were incubated with radiolabeled oligonucleotides corresponding to GATA/EBox wt as well as antibodies against MyoD, USF-1 and c-Myc which are known to bind to the EBox sequence. FP: free probe, 25×, 50×, 100× CO: 25-fold, 50-fold, 100-fold molar excess of cold oligo, No Ab: No Antibody, SRF: SRF antibody, FKHR: Forkhead antibody, GATA-4: GATA-4 antibody, c-Myc: c-Myc Antibody, USF-1: USF-1 antibody. **The representative blot of IRS/DNA binding was made from parts of the same gel. D. Representative chromatin immunoprecipitation from cells treated with or without 1 mM AICAR for 24 hours. Protein/DNA complexes were immunoprecipitated with USF-1 antibody, or with non-specific IgG. Primers encompassing the region between −473 and −823 were used to analyze USF-1 binding to the PGC-1α promoter. The representative blot on the left was made from parts of the same gel. At right is a graphical summary of repeated experiments. Values are representative of means±S.E.M (n = 6).

The responsiveness of the PGC-1α promoter to AICAR suggested that AMPK activation could mediate at least part of the increase PGC-1α mRNA expression via transcriptional activation. Since the largest portion of the activation effect occurred between −474 and −823 bp of the promoter (Fig. 2C), we evaluated the DNA binding activities of proteins bound to the putative consensus sequences within this region using gel shift analyses. The binding sites for the transcription factors found within this AICAR-responsive region of the promoter are shown in Fig. 1. They include an overlapping EBox/GATA sequence, an IRS sequence that binds FKHR [16], as well as an SRE and an EBox. AICAR treatment had no effect on the binding of proteins bound to the EBox, SRE, or the IRS sequences (Fig. 3A). However, AICAR treatment led to an ∼2.0-fold increase (p<0.05; Fig. 3B) in GATA/Ebox DNA binding. A number of possible candidate proteins exist which could bind to this overlapping sequence, a GATA isoform (i.e. GATA-4), c-myc, Upstream Stimulatory Factor (USF-1) or MyoD [30]–[32]. Analyses using specific antibodies against GATA-4, c-myc and MyoD did not reveal a supershift in the GATA/EBox-DNA complex (Fig. 3B and C). However, a strong supershifted complex was apparent in the presence of the USF-1 Ab (Fig. 3C), suggesting that USF-1 is a protein bound to the EBox within this sequence. The physiological relevance of this finding *in vivo* was evaluated with the use of ChIP analysis in which we found USF-1 bound to the PGC-1α promoter in non-stimulated conditions (Fig. 3D, lane 3). Following 24 hours of AICAR treatment, the amount of USF-1 bound to the PGC-1α promoter *in vivo* was increased by 2.3-fold (Fig. 3D, lane 5), consistent with the gel shift analysis above.

**Figure 4:**
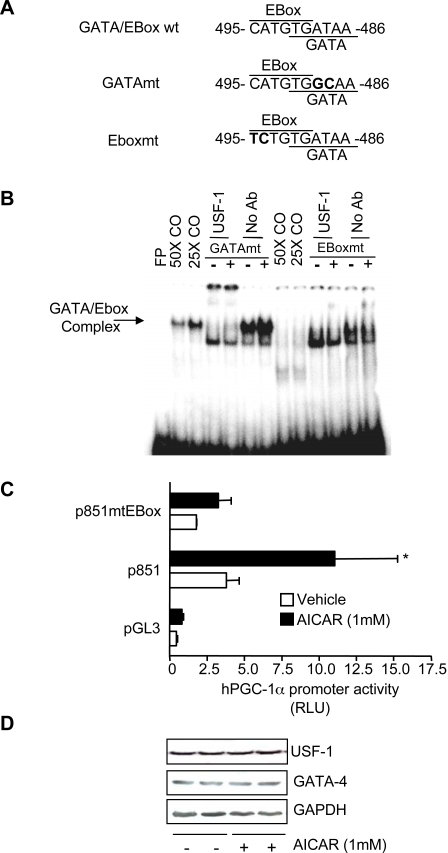
Mutations to either GATA or EBox elements alters AICAR-induced increases in GATA/EBox DNA binding and affects PGC-1α promoter activity. A. A schematic of the overlapping EBox/GATA (GATA/EBoxwt) binding sites at −495 and −486 base pairs within the PGC-1α promoter is shown. Mutations to the GATA/EBoxwt oligonucleotide that were introduced by nucleotide exchange to abolish GATA (GATAmt) or EBox (EBoxmt) binding are bolded. B. Representative EMSAs of Vehicle- (−) and AICAR− (+) treated nuclear extracts incubated with GATAmt or EBoxmt radiolabeled probes in the presence or absence of USF-1 Antibody. FP: free probe, 25× CO, and 50CO: 25-fold and 50-fold molar excess of cold oligonucleotide, No Ab: No Antibody, USF-1: USF-1 antibody. C. Relative luciferase activity of the PGC-1α promoter constructs in vehicle- or AICAR-treated cells is shown. Values are means±S.E.M, * p<0.05 versus Vehicle-treated control (n = 3). D. Western blot analysis of GATA-4 and USF-1 protein expression from total proteins extracted from vehicle− (−) and AICAR (+)-treated cells is shown. GAPDH expression was used as a loading control.

We then tested whether AMPK activation targets the EBox, the GATA sequence, or both sequences to transcriptionally activate the PGC-1α promoter by generating mutations within the EBox/GATA oligonucleotides to abolish either Ebox or GATA binding separately (Fig. 4A). Mutation of the GATA sequence shifted the DNA binding complex to a lower molecular weight, consistent with the absence of GATA-4 (Fig. 4B, lane 4–5 from the left). Although USF-1 was still present in the complex, the AICAR effect on protein-DNA binding was reduced (lane 7). However, when the EBox was mutated, USF-1 binding was prevented (lanes 10, 11), and the AICAR effect was abolished (lane 13). These data suggest that transcription factor binding to the EBox sequence is more important to mediate the effect of AMPK activation, but also suggest the fact that GATA could modulate the AMPK effect on DNA binding in regulating the activity of the promoter. This is suggested by the results showing that GATA-4 overexpression alone increased the transcriptional activity of the PGC-1α promoter (see below). Although the identity of the specific GATA isoform bound to the GATA sequence remains unresolved, GATA-4 is expressed in striated muscle [33], which suggests the possibility that the GATA-4 isoform is the likely candidate to mediate transcription of the PGC-1α promoter at this site.

The importance of the EBox as the primary mediator of the AICAR effect on PGC-1α promoter activity was further evaluated by generating a reporter construct in which mutations were incorporated into the Ebox sequence of the overlapping GATA/EBox sequence within the p851 promoter length. This mutation effectively prevented EBox protein/DNA binding. As shown in Fig. 4C, the typical effect of AICAR on the p 851 promoter length was abolished by a mutation of the EBox within the overlapping GATA/EBox. In addition, basal PGC-1α promoter activity was also reduced. These data, together with the DNA binding assays, suggest that USF-1 regulates both basal and AICAR-induced PGC-1α promoter activity.

To evaluate whether the increase in DNA binding observed in the presence of AICAR was the result of increased protein expression, we measured USF-1 and GATA-4 protein by western blotting. As illustrated in Fig. 4D, AICAR had no effect on either GATA-4 or USF-1 protein levels. Thus, the increase in DNA binding is likely attributed to AICAR-mediated post-translational modifications (i.e. phosphorylation) that may either directly or indirectly affect the transcriptional activity of GATA-4 or USF-1. There are numerous possibilities to explain the manner in which these modifications could occur. These include the direct AMPK-mediated phosphorylation of either GATA-4 or USF-1. Alternatively, the effect could be indirect and occur at multiple steps downstream of AMPK activation. Since the cellular signals and regulatory pathways that influence PGC-1α gene expression are not yet well understood, the elucidation of the underlying molecular mechanisms regulating PGC-1α gene transcription in response to AMPK activation are worthy of future study.

### Effect of GATA-4 and USF-1 overexpression on PGC-1α transcription and mRNA expression

**Figure 5:**
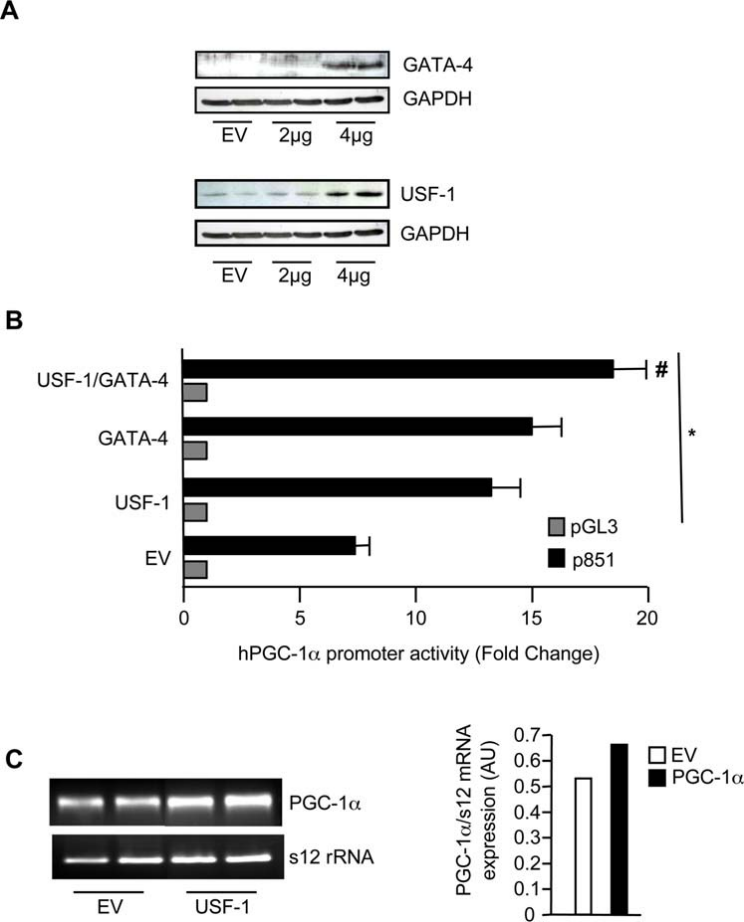
Effect of GATA-4 and USF-1 overexpression on PGC-1α promoter activity and mRNA expression. A. Representative western blots of protein extracts made from C_2_C_12_ cells transfected with either 2 or 4 µg of GATA-4 or USF-1 or an empty vector (EV) control. B. USF-1 and GATA-4 were co-transfected with the pGL3 (EV; 500ng) or the p851 PGC-1α promoter reporter construct (500ng) along with the appropriate empty vector controls. Relative luciferase activities were measured 48 hours after transfection and are plotted as the fold change above empty vector. Values are means±SEM, (n = 8); * p<0.05 versus p851-EV and #, p<0.05 versus p851-USF-1 or p851-GATA-4. C. Cells were transfected with 4 µg of USF-1 or an empty vector (EV) control. EtBr-stained DNA gel of PGC-1α amplified by PCR from EV- and USF-1 transfected cells. s12rRNA was used to verify equal loading. Data are representative of one experiment with conditions repeated in duplicate (AU = arbitrary units).

We sought to identify whether GATA-4 and USF-1 could mediate PGC-1α transcription independently. Thus, we overexpressed GATA-4, USF-1 or the two in combination in muscle cells. Increases in the levels of GATA-4 or USF-1 protein were detected by Western Blotting in cells transfected with 4 µg of DNA (Fig. 5A). USF-1 overexpression alone increased promoter activity by 1.7 fold (p<0.001), in addition to producing an increase in PGC-1α mRNA (Fig. 5C). The enhanced effect of USF-1 overexpression on promoter activity was not observed when the p851mtEbox was transfected, indicating that the USF-1 effect occurs via the Ebox element (data not shown). GATA-4 overexpression led to a 2.0- fold increase in promoter activity (p<0.05; Fig. 5B), but did not lead to increases in PGC-1α mRNA. This latter result was expected, since the GATA element found within the human promoter is not conserved in the mouse. The combination of USF-1 and GATA-4 overexpression produced a further significant increase in PGC-1α promoter activity to 2.5-fold above the empty vector control. The magnitude of this increase is suggestive of an additive effect of the two proteins on PGC-1α transcription.

## Discussion

PGC-1α regulates many important aspects of skeletal muscle biology including the stimulation of mitochondrial biogenesis and cellular respiration, muscle fiber type transitions, glucose metabolism as well as fatty acid oxidation [7], [27], [34], [35]. Significant health benefits can be derived from improvements in any of these factors in healthy individuals, and also in aging, obese, and/or physically inactive populations in which mitochondrial content or substrate metabolism is compromised. Thus, research efforts dedicated to the elucidation of the cellular signals and the regulatory pathways that influence PGC-1α gene expression are warranted, particularly under circumstances in which mitochondrial biogenesis is induced.

Several studies have established that endogenous PGC-1α gene expression is increased following contractile activity and thyroid hormone treatment in skeletal muscle [8]–[10], [23], [29], [36]–[38]. In addition, some of the cellular signals arising from muscle contraction that could account for this induction have also been recently identified. They include changes in intracellular Ca^2+^, and/or alterations in cellular energy status that activate AMPK [9], [18], [39], [40]. AMPK activation either during contractile activity, or with the use of the AMP analogue AICAR, has been associated with greater mitochondrial enzyme activities and PGC-1α expression in a variety of different experimental models [9], [18], [28], [40]. However, the detailed molecular mechanisms, as well as the direct effect of AMPK on PGC-1α gene transcription, have not yet been established. Thus, we employed the pharmacologic activation of AMPK in order to more precisely define the regulatory pathway which leads increased PGC-1α mRNA expression in skeletal muscle.

We show here that the use of AICAR for 24 hours activates AMPK and increases PGC-1α mRNA expression, a finding that is similar to that observed by others using different experimental models [27], [37], [40]. Since the underlying cause of this induction is proposed to be linked to a transcriptional mechanism [13]–[17], we cloned a proximal sequence of the PGC-1α promoter and analyzed this fragment with stringent computer-assisted analyses to identify candidate transcription factor binding sites that could mediate the AICAR effect. This 2.2 kb sequence was found to contain putative binding sites for transcription factors that are typically found within the regulatory regions of muscle-specific genes [6], [41]–[45]. To our knowledge, this represents the first extensive documentation and characterization of the human PGC-1α promoter, other than the identification of the genomic organization and chromosomal location of the *pgc-1*gene by Esterbauer et al., [24]. Interestingly, none of the putative binding sites that were identified by our analysis had been previously identified as AMPK targets. Thus, we generated a series of 5′-deletion constructs to test the general responsiveness of the promoter to AICAR and also to identify the regulatory regions responsible for regulating PGC-1α transcription.

Our data show that both AICAR-positive and -negative regions are present within the 2.2 kb sequence. This is highlighted by the fact that not all PGC-1α promoter lengths were positively affected by AICAR. This suggests that there are several putative binding sites for transcription factors that confer a negative effect on PGC-1α promoter activity. These data therefore establish that a combinatorial interplay between positive and negative stimuli ultimately act on the PGC-1α promoter to upregulate PGC-1α mRNA expression. Since endogenous PGC-1α mRNA expression was increased in response to AICAR, we sought to elucidate mechanisms which could account for the effects of this AMP analogue. The two AICAR-positive regions that were identified suggested the possibility that AMPK may target the CRE or other elements within the p 191 promoter as well as the binding sites found at −823 to −473. However, two pieces of evidence suggest that the latter region accounts for the majority of the responsiveness to AICAR. First, the AICAR-responsive region between −823 and −473 is sufficient to transcriptionally activate a minimal promoter in response to AICAR. Second, gel shift experiments and promoter assays using oligos, or using the 851 promoter length in which the EBox of the overlapping GATA/EBox was mutated, completely abolished the AICAR effect. These findings highlight an unexpected appreciation for the complexity that underlies the regulation of PGC-1α promoter activity, given the recent work by Akimoto et al., [14] in which they demonstrated that the exercise-induced activation of PGC-1α transcription in muscle relies exclusively on a distal MEF and a proximal CRE sequence. Based on their findings, we anticipated that AMPK activation by AICAR would elicit an increase in the activity of the promoter fragment containing either the MEF2 site (i.e. p 2218) and/or the CRE site (p 191), since AMPK is activated both by exercise [20]–[22], as well as by electrically-evoked contractions in C_2_C_12_ cells [9]. Thus, our experimental approach to assess PGC-1α promoter regulation using a variety of deletion constructs has allowed us to characterize additional regulatory elements that were unidentifiable using only one promoter length.

Further characterization of the DNA sequences that lie upstream of 2.2 kb are also likely to reveal additional regulatory regions, since the relative effect of the AICAR response with respect to promoter activation (3.5-fold) and mRNA expression (2.2-fold) in this study were not of equal magnitude. For example, an important distal MEF2 site, identified at −2901 bp in the mouse promoter [15], may be involved. In the human PGC-1α promoter, this MEF2 site is found at position −3344, and may have a similar function in the regulation of PG-1α expression. In addition, several alternative explanations that may account for the differences in the responses to AMPK activation, including alterations in mRNA stability and a divergence in the mechanisms governing the regulation of the endogenous versus the ectopically expressed promoter, were considered. Our preliminary observations indicate that the AICAR-mediated increase in PGC-1α mRNA expression is not the result of enhanced mRNA stability (Irrcher and Hood, unpublished observations). However, some of the differences in response to AICAR may be explained in part by the protective effect of histones surrounding the endogenous promoter. This argument is supported by the recent report that the *in vivo* regulation of the PGC-1α promoter by HDAC5 is a key mechanism influencing endogenous PGC-1α expression in the heart [15].

We evaluated a role for GATA and Ebox binding proteins in the direct regulation of PGC-1α promoter activity and mRNA expression. Supershift analyses clearly identified USF-1 as a protein bound to the overlapping GATA/EBox sequence, which was confirmed *in vivo* using chromatin immunoprecipitation. Furthermore, overexpression of USF-1 increased PGC-1α promoter activity and led to a similar induction in PGC-1α mRNA expression. GATA-4 overexpression also increased PGC-1α promoter activity. Because mutation of the GATA element failed to reduce DNA binding in electromobility shift analyses (Fig. 4B), and because mutation of the Ebox reduced the AICAR effect to that of control values (Fig. 4C), we believe that PGC-1α expression is more likely to be regulated by USF-1 rather than GATA-4 in response to AICAR. Although the Ebox within the overlapping sequence mediates the AICAR effect on PGC-1α promoter activation, the fact that AICAR also induces the activation of the p191 reporter construct suggests that the mRNA response may also rely on the cooperative actions between USF-1, GATA-4 and other transcription factors bound to neighboring or more distal sites, for the complete AMPK-mediated induction of the promoter.

In summary, we describe the transcriptional activation of the PGC-1α promoter in response to AMPK activation. Although it cannot be definitively be ruled out that AICAR may have unknown effects other than AMPK activation, our study clearly demonstrates that the use of this activator is associated with an increase in AMPKα phosphorylation in Thr172, and that this phosphorylation coincides with the associated changes in PGC-1α gene transcription. At least one important mechanism that may account for this effect is the enhanced GATA/EBox DNA binding, an effect that is primarily mediated by USF-1. The activation of the PGC-1α promoter can also be stimulated by the independent actions of USF-1 and GATA-4. Thus, we have described potential novel AMPK transcription factor targets which further contribute to the elucidation of the mechanisms that underlie the induction of PGC-1α in skeletal muscle. A complete understanding of the regulation of PGC-1α expression in skeletal muscle has potential therapeutic value, since low levels of this coactivator are associated with a disruption of energy homeostasis [11], [46] and the manifestation of diseases such as insulin resistance and diabetes [12], [47].

## Materials and Methods

### Chemicals and Reagents

5-aminoimidazole-4-carboxamide-1-β-D-ribofuranoside (AICAR) was purchased from Calbiochem (La Jolla, CA). AICAR was resuspended in sterile double distilled H_2_0 at 50 mg/ml and stored at −20°C. Nitrocellulose membrane and [γ-^32^P] dATP were obtained from GE Health Care (Baie D'Urfé, Quebec). Restriction enzymes, phospho-AMPKα (T172; Cat.No.2531) and AMPKα (Cat.No.2532) antibodies were from New England Biolabs (Mississauga, Ontario). The pGL3-basic and pGL4.23 (*luc2/*minP) reporter vectors as well as the dual luciferase assay system were from Promega (Madison, WI). Lipofectamine 2000 and SuperscriptII first strand cDNA synthesis kit was obtained from Invitrogen (Burlington, Ontario). Cell culture reagents were purchased from Sigma (St. Louis, MO). Synthetic oligonucleotides and PCR primers were from Sigma Genosys (Toronto, Ontario). Antibodies against GATA-4 (H-112), and USF-1 (H-86), c-myc (C-33), SRF (G-20), and MyoD (C-20) were purchased from Santa Cruz (Santa Cruz, CA). The GAPDH antibody (ab8245-100) was from Abcam (Cambridge, MA).

### Cell culture and treatments

C_2_C_12_ muscle cells were cultured as previously described [9]. Briefly, cells were maintained in DMEM containing 10% FBS and 1× Antibiotic/Antimycotic. When cells reached 90% confluence, they were switched to DMEM containing 5%-heat inactivated horse serum and 1× Antibiotic/Antimycotic and treated for 24 hours with either vehicle or AICAR (1 mM), to activate AMP kinase [48].

### PGC-1α promoter cloning and construction of plasmids

A 2.2 kb fragment of the human PGC-1α promoter was generated by PCR using genomic DNA isolated from skin fibroblasts as a template. PCR primers were designed from the PGC-1α promoter sequence (Gen Bank Accession Number BD 103728; sense: 5′-GCT GGT ACC GTG TCC AGT ACC TTG AGT TTG -3′, antisense: 5′-ACA CTC ATG CAG GCA ACC AG-3′). The cloned fragment was ligated into the *KpnI*- and *HindIII*- digested sites of the pGL3-basic vector containing the luciferase reporter gene. This fragment (p 2218) contains 2190 base pairs 5′- and 28 base pairs 3′ relative to the transcription start site and served as a template to generate 5′-serial deletions of the promoter. The resulting PCR-amplified fragments containing 1136, 823, 473, and 163 base pairs 5′- and 28 base pairs 3′- relative to the transcription start site, were ligated into *KpnI*- and *HindIII*- digested sites of the pGL3-basic vector containing the luciferase reporter gene. These constructs generated plasmids p 1164, p 851, p 501, and p 191, respectively. The p851ΔEbox reporter construct containing a CA→TC mutation to prevent Ebox protein/DNA binding was generated by PCR. The AICAR-responsive region (ARR) was cloned into the *Kpn1-* and *HindIII*- digested sites of the pGL4.23 promoter reporter vector containing a minimal promoter. All plasmids were verified by sequencing.

### Luciferase reporter assay and transient transfections

Where indicated, C_2_C_12_ cells were cultured in 6-well dishes and transiently transfected with 500 ng of the reporter plasmids, GATA-4 (2 µg or 4 µg) and/or USF-1 (2 µg or 4 µg) using Lipofectamine 2000, following the manufacturer's recommendations. The amount of DNA in each transfection was kept constant by the addition of the appropriate empty vector (EV) controls. Transfection efficiency was normalized to Renilla luciferase activity (pRL-CMV; 5 ng/plate). Following treatments, cell extracts were prepared using 1×Passive Lysis Buffer. Luciferase activities were measured using an EG&G Berthold (Lumat LB9507) luminometer, according to the manufacturer's instructions. The GATA-4 and USF-1 expression plasmids have been described elsewhere [49], [50].

### Nuclear extract preparation and Electrophoretic mobility assays (EMSA)

Nuclear extracts used in EMSAs were as done previously described [51]. Briefly, extracts (5-15 µg) were incubated with 20 µg/ml poly(dI-dC), 50 µM PPi and 40,000 CPM of [γ-^32^P] dATP end-labeled oligonucleotides containing putative DNA consensus sequences (in bold) within the −473 and −823 bp region of the hPGC-1α promoter corresponding to the EBox (5′-CTA ACA CTT GTT TTC TGG GAG C-3′), SRE (5′-GGA TGT CCA TAT TAG GAC ATC CT-3;), IRS (5′-TAC AGC CTA TTT TGT TGA TTA A -3′), GATA/EBox (5′-GCA TCA TGT GAT AAG GCT CCT GC-3′), GATAmt in which the GATA sequence was mutated (mutations are underlined; 5′-GCA TCA TGT GGC AAG GCT CCT GC-3′), and EBoxmt in which the EBox sequence was mutated (mutated are underlined; 5′-GCA TTC TGT GAT AAG GCT CCT GC-3′). Samples were run on native 5% acrylamide gels for 1.5 hours at 250 V. Gels were subsequently fixed for 15 mins (acetic acid/methanol/water (10∶30∶60), dried and imaged using an instant imager (Packard).

### Chromatin Immunoprecipitation (ChIP)

ChIP assays were conducted using the ChIP-IT Express Kit (Active Motif) according to the manufacturer's instructions. Briefly, cells in three 150 mm plates per condition were crosslinked with 1% formaldehyde for 10 mins at room temperature. The reaction was stopped with the addition of Glycine Stop-Fix solution, and then washed with ice-cold 1×PBS. Cells were pooled, pelleted and then incubated on ice for 30 mins in 1× Lysis Buffer supplemented with 100 mM PMSF and Protease Cocktail Inhibitor Mix. Cells were then transferred to an ice-cold dounce homogenizer and homogenized on ice with 40 strokes to aid in the release of nuclei. Following sonication (35 pulses, 20 sec/pulse at 25–30% power) and centrifugation, 12 µg of sheared chromatin was incubated with magnetic-coupled Protein G beads, anti-USF1 or IgG (as negative control) overnight at 4°C. An aliquot of chromatin that was not incubated with an antibody was used as the input control sample. Antibody-bound protein/DNA complexes were washed, eluted, and treated with Proteinase K to digest proteins. The chromatin was then used in PCR analyses. Primers amplifying the mouse PGC-1α promoter were: F: 5′AGC TGA TCT GAG CAG AGC AG-3′ and R: 5′-CTC AAG CTC AGT TTG GGA CT-3′, which generated a 543 bp product. PCR products were resolved on 1.8% agarose gels containing ethidium bromide. Gels were scanned and quantified with SigmaGel software (Jandel).

### Reverse transcription PCR

Total RNA from vehicle- and AICAR-treated C_2_C_12_ cells was isolated using TRIzol reagent, following the manufacturer's recommendations. The purity and concentration of total RNA was determined spectrophotemetrically. Equal amounts (0.5 µg) of total RNA were reverse transcribed using Superscript II with Oligo-18(dT) as primer, following the manufacturer's recommendations. Sequence-specific primers to amplify PGC-1α (5′-GAC CAC AAA CGA TGA CCC TCC-3′ (F) and 5′-GCC TCC AAA GTC TCT CTC AGG-3′ (R) and GAPDH 5′-TGC TGA GTA TGT CGT GGA GTC TA-3′ (F) and 5′-AGT GGG AGT TGC TGT TGA AGT CG-3′ (R) were used, yielding product sizes of 635 bp and 602 bp, respectively [52]. PCR reactions were carried out in a 50 µl volume containing 2 µl cDNA, 10 µl 5× *GoTaq*DNA Buffer, 0.2 mM dNTPs, 3 mM MgCl_2_, and 1 unit of *Taq* DNA polymerase. Total RNA samples were also tested without the addition of reverse transcriptase to verify the absence of genomic DNA contamination. The conditions for both primer sets included an initial denaturing step (94°C at 2.5 mins). PGC-1α was then amplified using the following conditions: denaturation at 94°C for 30 sec, annealing at 69°C for 30 sec, and extension at 72°C for 30 sec for 29 cycles with an additional 5 minute extension time. GAPDH was amplified using the following conditions: denaturation at 94°C for 30 sec, annealing and extension at 72°C for 30 sec for 23 cycles with an additional 5 minute extension time. Optimal cycle number was determined to obtain a PCR product within the linear range. PCR products were resolved on 1.8% agarose gels, scanned and quantified with SigmaGel software (Jandel).

### Western Blotting

Total protein was isolated from C_2_C_12_ cells as done previously [9]. Briefly, total protein (20–40 µg) was electrophoresed through SDS-polyacrylamide gels and transferred onto nitrocellulose membranes. The membranes were subsequently probed overnight with antibodies directed toward GAPDH (1∶20,000), GATA-4 (1∶1,000), USF-1 (1∶500), phospho-AMPK_α_ (1∶400), or AMPK_α_ (1∶1,000), washed 3×5 mins with TBS-Tween20, incubated for 1 hour at room temperature with the appropriate secondary antibodies conjugated to horseradish peroxidase, visualized with enhanced chemiluminescence, and quantified using SigmaScanPro (Jandel, San Rafael, CA).

### Statistics

All data are expressed as means±S.E.M. Where indicated, Students' unpaired t-test or 2-way ANOVAs followed by Bonferroni post-hoc tests, respectively, were used to determine individual difference between conditions. Results were considered to be statistically significant if p<0.05 was achieved.
